# Advancing urban health equity in the United States in an age of health care gentrification: a framework and research agenda

**DOI:** 10.1186/s12939-022-01669-6

**Published:** 2022-05-11

**Authors:** Helen V. S. Cole, Emily Franzosa

**Affiliations:** 1 Barcelona Lab for Urban Environmental Justice and Sustainability, Institut de Ciencia i Tecnologia Ambientals (ICTA-UAB), Universitat Autonoma de Barcelona, Barcelona, Spain; 2 Healthy Cities research group, Department of Epidemiology and Public Health, Institut Hospital del Mar d’Investigacions Mèdiques (IMIM), Barcelona, Spain; 3 Research Health Science Specialist, Geriatric Research Education and Clinical Center (GRECC), James J. Peters VA Medical Center, Bronx, USA; 4 Brookdale Department of Geriatrics and Palliative Medicine, Icahn School of Medicine at Mount Sinai, New York, USA

**Keywords:** Health care access, Gentrification, Health equity, Urban health

## Abstract

**Background:**

Access to health care has traditionally been conceptualized as a function of patient socio-demographic characteristics (i.e., age, race/ethnicity, education, health insurance status, etc.) and/or the system itself (i.e., payment structures, facility locations, etc.). However, these frameworks typically do not take into account the broader, dynamic context in which individuals live and in which health care systems function.

**Purpose:**

The growth in market-driven health care in the U.S. alongside policies aimed at improving health care delivery and quality have spurred health system mergers and consolidations, a shift toward outpatient care, an increase in for-profit care, and the closure of less profitable facilities. These shifts in the type, location and delivery of health care services may provide increased access for some urban residents while excluding others, a phenomenon we term “health care gentrification.“ In this commentary, we frame access to health care in the United States in the context of neighborhood gentrification and a concurrent process of changes to the health care system itself.

**Conclusions:**

We describe the concept of health care gentrification, and the complex ways in which both neighborhood gentrification and health care gentrification may lead to inequitable access to health care. We then present a framework for understanding health care gentrification as a function of dynamic and multi-level systems, and propose ways to build on existing models of health care access and social determinants of health to more effectively measure and address this phenomenon. Finally, we describe potential strategies applied researchers might investigate that could prevent or remediate the effects of health care gentrification in the United States.

## Background

The 2019 closure of Hahnemann University Hospital in Philadelphia’s rapidly gentrifying city center following its sale to a private equity investor captured national attention. The safety-net hospital, which served a primarily Black, publicly insured and uninsured patient population, was rumored to be replaced with a more profitable real-estate venture. In the ensuing months, the impact of Hahnemann’s closing was felt deeply as neighboring hospitals absorbed increased emergency traffic [1], and Black Philadelphians who had relied on the hospital were hard hit by the COVID-19 pandemic [2]. While Hahnemann’s was an especially high-profile closure, it is only one of many across the United States (U.S.) that represent a broader, complex shift in the type, location and delivery of urban health care services that we term *health care gentrification*.

Just as urban gentrification brings amenities and services for new, affluent residents to formerly underinvested neighborhoods, health care gentrification in U.S. cities, occurring concurrently and in addition to neighborhood gentrification, provides increased access to health care for some residents while excluding others – primarily minority, low-income, and uninsured or publicly insured patients. For instance, while safety-net hospitals like Hahnemann in gentrifying areas are increasingly closing due to financial pressures, these same neighborhoods are seeing an influx of urgent care centers and specialty outpatient practices targeted to privately insured patients [3]. Our emerging research in four U.S. cities suggests that health care gentrification, exacerbated by socio-spatial inequities made worse by neighborhood gentrification, may be driving inequities in health care access and, in turn, population health.

In this essay, we argue that research on urban health care access must be considered within a broader urban planning framework, taking into account broader changes occurring in cities that drive health care gentrification. We start from the understanding that health care systems do not exist in isolation but are part of complex urban systems. We focus on the city-scale because cities themselves experience a high degree of socio-spatial inequities in health, are unique in that they are often tasked with ensuring the health of residents, yet lack control of health care systems which, in the U.S., consist largely of private entities governed by multiple levels of state and federal laws, regulations and payment mechanisms [4, 5].

Our framework considers access to high-quality health care services to be a critical resource for healthy and equitable neighborhoods, and one that may be threatened by health care gentrification [6, 7]. To better understand the complex interplay of factors affecting access to health care, we first describe the relationship between neighborhood and health care gentrification and its potential impact on accessing and providing high quality health care. We then present a framework for understanding health care gentrification as a function of dynamic and multi-level systems and propose ways to build on existing models of health care access and social determinants of health to more effectively measure and address this phenomenon. Finally, we describe potential strategies to prevent or remediate the effects of health care gentrification.

### Neighborhood gentrification and urban health equity

Gentrification is a process of neighborhood change through which the demographic, real estate, and business characteristics of a place transition toward a population that is wealthier, whiter, has a higher level of formal education, and is able to afford new or renovated, more expensive homes while also fomenting new cultural and consumption practices [8]. Neighborhood gentrification is closely linked to patterns of uneven development and deprivation which have long been understood as social determinants of health and to lead to health inequities manifested as differences in health outcomes between residents of different types of neighborhoods. Studying the health effects of gentrification goes beyond more traditional research on neighborhood health effects by highlighting how neighborhoods change over time, and how these changes may impact social and health inequity among communities residing in the same neighborhood environments [9].

Emerging research suggests that while gentrification may be beneficial for the health of dominant racial, ethnic or class groups (so called “gentrifiers”), it can harm that of ethnic or racial minorities (particularly Black residents) or lower socioeconomic classes, who are often long-term residents, exacerbating health inequalities [10, 11]. There are a number of hypothesized mechanisms by which neighborhood gentrification may affect health, including changes in neighborhood social dynamics, social networks and support; exclusion of long-term or lower income residents from health promoting neighborhood resources (such as parks, healthy food stores, and others); increased instances of violence and insecurity; and the stress of threat of or actual displacement and housing instability [12, 13]. The complexity of neighborhood social environments also has significant implications for the provision of high-quality health care. Health care providers struggle to diagnose and treat patients facing complex social conditions such as homelessness, inadequate housing, exposure to violence, and others [14], and neighborhood gentrification may exacerbate these issues, particularly for marginalized residents. For instance, one study found that residents displaced from gentrifying neighborhoods were more likely to visit emergency rooms for mental health-related reasons and to be hospitalized for mental health issues [6].

### Health care gentrification and urban health equity

While research on neighborhood gentrification and health often focuses on the health impacts of gentrification on residents, *health care gentrification* refers specifically to the impact of changing distribution of health care resources within a community on residents’ abilities to access health care they need, taking into account differences in financial means and health needs by social group. Much in the same way that income inequality and consistent underinvestment in certain neighborhoods create conditions in which neighborhood gentrification flourishes, inequities in income and wealth, insurance status, and privatization of services put health care resources at risk for gentrification. Our interest is in understanding what these changes mean for equity in accessing health care.

Within the U.S. health care system, the location of services is driven by multi-level factors, including a mix of federal and state policies and regulations (for instance, siting federally-qualified health centers, or “FQHCs”, that are charged with delivering community-based care in areas of highest need) and payments from insurers and individuals [15]. Within this system, the growing trend toward more efficient, profitable, and value-based care that rewards “quality” over quantity of services has incentivized providers in new ways [16, 17]. As private health care systems increasingly consolidate and acquire hospitals, provider practices, and services like labs and radiology centers [18], they also capture more revenue and geospatial advantage. From a health care gentrification standpoint, when large private “megasystems” consolidate, it makes business sense to shutter underperforming hospitals, close less profitable service lines such as maternity or emergency care, and invest in higher-profit specialty practices such as cardiology or neurology. The shift toward outpatient care has also proven a lucrative opportunity for private equity investors, who have put significant resources into health care ventures for profit [19, 20].

In addition to offering more profitable types of care, comparatively low reimbursement from public government-funded insurance such as Medicare and Medicaid drives health care providers to seek out more profitable patients by prioritizing the needs and preferences of the commercially insured. “Payer mix”, or the proportion of patients insured privately through commercial health plans, is a strong driver of the distribution of health care services geographically [21]. Efforts to improve payer mix by siting outpatient and specialty services in gentrifying areas may leave uninsured or publicly insured residents without a source of care, even though these patients are more likely to have urgent or severe health needs, and depend on neighborhood providers because of limited mobility or access to transportation [22]. In addition, demands (and ability to pay) for different types of services and the desire to avoid wait-times may also drive providers to create new settings such as “walk in” clinics, free-standing emergency rooms, or concierge care providing on-demand direct access to physicians, at a price [23, 24]. This means private providers may siphon off more profitable residents, leaving overburdened and underfunded public and charitable systems to fill the gap.

The location of health care services is only one facet of health care access, and in turn, distributional equity [25]. To maximize revenue and minimize loss, health systems can also restrict access by changing the supply of care by limiting capacity, refusing patients, charging higher prices, or not providing culturally or linguistically appropriate providers [26–28]. For instance, many proprietary urgent care clinics do not accept Medicaid, the public health insurance for lower-income individuals [29, 30]. Policy and regulations can also limit provider supply; while the Affordable Care Act was designed to increase access to health insurance, differences in the adoption and implementation of health care exchanges and Medicaid expansion have exacerbated inequity between states, and often by race and social class [31, 32].

### A health care gentrification framework

**Fig. 1:**
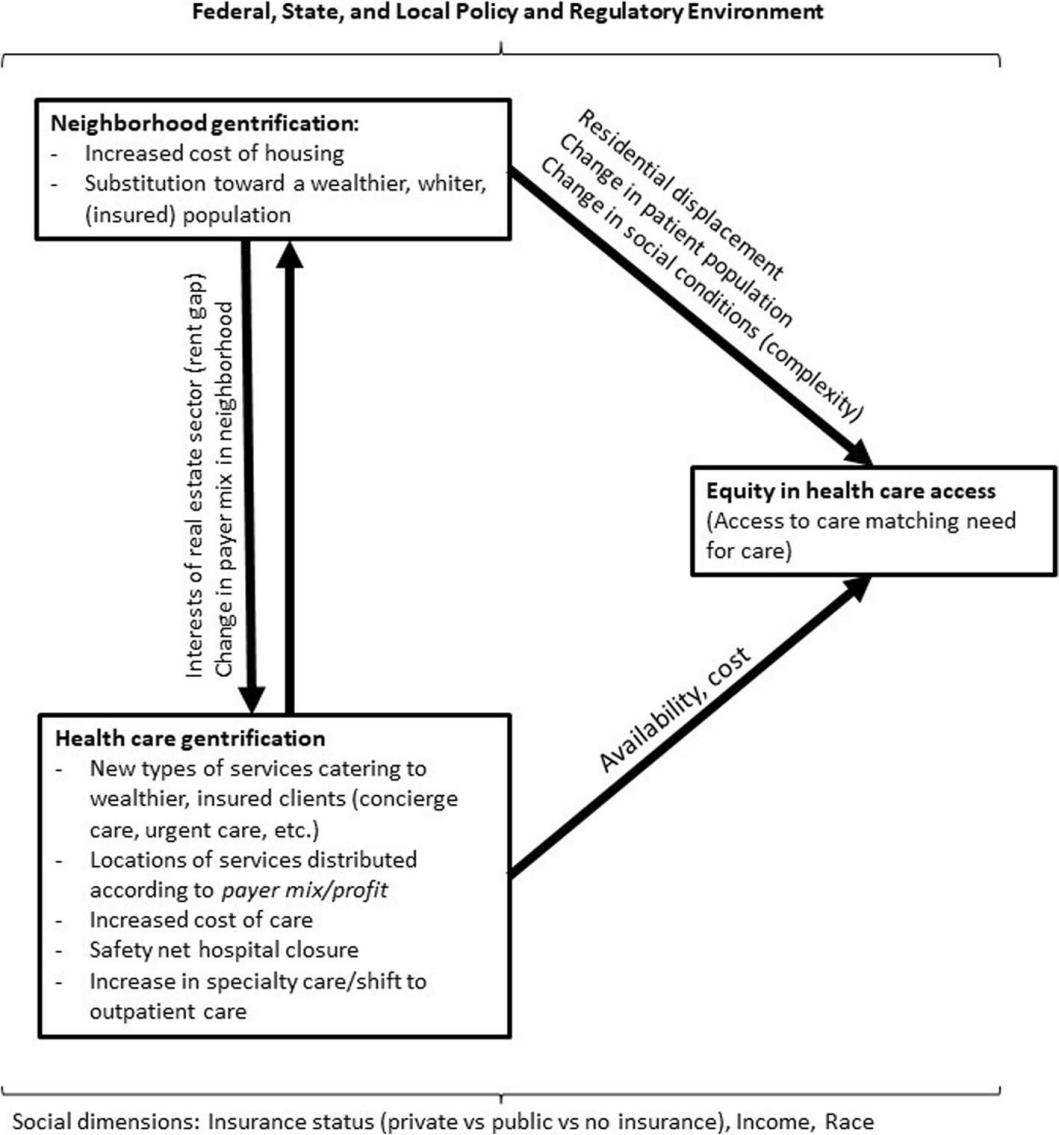
The relationship between neighborhood gentrification, health care gentrification, and equity in health care access

Placing health care access within the context of neighborhood environments, we present the health care gentrification framework (Fig. 1) to guide the formation of new research questions and methods. We conceptualize access to health care as a function of changing social and physical neighborhood environments over time, and as a function of the dynamic health care system, both of which are influenced by policies, regulations, and private actors at the national, state and local level. Thus, we frame the understanding of health care access within the context of inequities created by neighborhood gentrification, further exacerbated by inequities in access to care resulting from recent changes to health care systems.

As neighborhoods gentrify, residents may lose a usual source of care as they or their providers are displaced or have to travel further for care. But our research suggests more complex, and important, connections between neighborhood and healthcare gentrification as well. For instance, health care gentrification becomes entwined with neighborhood gentrification via real estate speculation. The “rent gap” [33] of a failing hospital is quite large. That is, a failing hospital could gain substantial profit if transferred to another use such as market-rate housing, and even more so if it is located in a gentrifying neighborhood where property prices are rising, as occurred with Philadelphia’s St. Joseph’s Hospital and Queens, New York-based St. John’s hospital, to name a few [34, 35].

The interaction between these neighborhood and health care factors may either promote or reduce access to health care services. Access to high quality health care is essential for maintaining health, providing lifesaving treatment for acute health conditions, injuries, illnesses, and both prevention of and ongoing treatment of chronic disease and disability. Access to care also plays a salient role in maintaining health equity within and among populations. We maintain that marginalization of certain social groups due to widespread poverty and systematic racism, entwined with neighborhood racial and class segregation, produce conditions in which both the burden of disease and access to care are not equitably distributed [36, 37]. Thus, to ensure equity in access to care, health care systems must distribute (and be incentivized to distribute) services based on these needs rather than profit and demand from privileged communities.

### Considerations for measuring health care gentrification

Measuring the processes and impacts of health care gentrification can start by building on decades of health services and public health research to identify the many factors determining access to health care. These include not only providers’ geographical locations and capacity in relation to the population and systems characteristics such as payment structures and gatekeeping policies, but also characteristics of patients themselves (i.e., socioeconomic status or language) and of health care systems (i.e., payment structures, locations, and management). Focusing on the U.S. health care system, Aday and Anderson, for example, measured both potential and realized access to care through utilization and outcome measures [38]; while Penchansky and Thomas framed access as the “fit” between patients’ need and the system’s ability to meet them through five dimensions: availability, accessibility, accommodation, acceptability, and affordability [39]. More recently, Fortney et al. extended this framework, proposing that “fit” should include geographical, temporal, financial, cultural and digital dimensions of access, as well as patients’ own perceptions of their access to care and need for care [40]. Levesque and colleagues similarly proposed incorporating patient dimensions, including the ability of individuals and populations to perceive, seek, reach, pay for and engage with the health care system [25].

However, operationalizing these models has been challenging and reveals limitations in measuring this complex and multi-dimensional construct, particularly from a *health equity* standpoint. In practice, studies of access often focus on proxies for utilization using cross-sectional analyses of national survey data; however, as Ricketts and Goldsmith have noted, these types of measures tend to relate to discrete events and outcome measures at the individual level, and relatively fixed variables at the population level, such as overall insurance coverage, making it difficult to identify broader trends and drivers, and to measure the impact of spatial, social and organizational changes over time [41]. In addition, many of these measures fail to account for the social and political structures driving access measures, highlighting the need for new and more expansive models that go beyond health services to examine broader systems. Cyr, for instance, suggests placing domains of access within a social ecological framework, and identifies emergent domains including the role of government and insurance policy (for instance, government-funded medical training programs that can redistribute medical students to underserved areas) and the influence of health organizations and operations, an important factor as health systems increasingly re-organize and consolidate [42]. Finally, a reliance on aggregate national survey data and geospatial data may overlook the lived experiences of individuals and communities.

Similarly, neighborhood gentrification research has both benefits and drawbacks to adding to our understanding of health care access. Research on gentrification and health has primarily employed quantitative measures derived from census and other data as proxies for gentrification, which are then considered as an exposure in epidemiological models measuring risk of specific or general health outcomes [9, 43]. Some have also considered the role of gentrification as a moderator in measuring the effects of other exposures such as the benefits of exposure to health promoting resources in cities [9], e.g., showing the benefits of access to green space, while improving health at a population level, may not reach lower-income or less educated residents [44]. A few studies have also used qualitative methods such as interviews, focus groups or photovoice to tease out the mechanisms by which gentrification may affect health, and particularly the health of long-term residents, and understand and translate community members’ experience of access into person-centered measures [7, 14, 45, 46].

A summary of potential measurements is included in Table 1. While existing cross-sectional measures and geospatial data are important and helpful in identifying broader trends, we believe that measuring the nuances of health care access must also include longitudinal data to understand dynamic change over time (such as those measures tracking gentrification, itself a change) and system-level measures at multiple levels (local, state and national) to identify the impact of broader political and policy structures on how health care resources are allocated. Data and methods to answer these questions may be drawn from related fields such as the environmental justice movement [47], which complements and expands on the more traditional health services and social determinants of health frameworks by joining concepts of access as a function of the distribution of services with a deeper look at the underlying political, governance and economic factors contributing to inequitable access to care and by conceptualizing health care itself as a part of the broader urban environment.

**Table 1:** Domains of health care gentrification, potential measures and data sources

Domain	Potential measures	Potential Data Sources
Health care utilization	Patient visits to health care facilities, receipt of screening services for chronic disease, vaccination rates, types of services and facilities used	Surveillance or survey data from city, state or national agencies; survey data; qualitative data from neighborhood residents
Health care access	Provider supply/population, location, types of insurance accepted, wait lists and wait times, cultural competency	Census and survey data, city and state health department surveillance data, Qualitative data from neighborhood residents
Neighborhood gentrification	Gentrification index; perceived gentrification measures; local knowledge and experiences	Census and survey data; qualitative data from neighborhood residents
Health care system	Health systems acquisitions, mergers and closures; staff layoffs/restructuring; opening of new facilities, practices and types of services	Media coverage; closure plans; local government hearings; qualitative research with health care system administrators and workers
Policy	Regulation of medical licensing, facilities and training; scope of practice laws	Federal, state and local laws and regulations; federal register; policy documents; government hearings; media coverage

### A health care gentrification research agenda

To ensure equitable access to quality health care in U.S. cities, there are several areas of investigation which deserve focus—primarily research into the causes, effects, and measurement of health care gentrification and broader urban processes which may affect who has access to high quality health care that meets their needs. Thus, here we consider how research on access to health care can be made more equity-centered by considering not only the characteristics of patients and providers/health care systems that predict access, but also the underlying drivers of provider/systems characteristics that lead to more or less equitable access to care. Drawing on an ecological model and Purnell et al’s work on factors influencing disparities in access to care, we propose research questions and interventions that these questions might examine at the policy/regulatory, organizational, community and individual level (summarized in Table 2). Many of these questions and interventions cross multiple levels, highlighting the importance of multi-level, mixed methods research and intervention targets [48].

**Table 2:** A sample of proposed healthcare gentrification research questions and interventions

Level of Inquiry	Potential Research Questions	Promising Interventions to Study
Policy/Regulatory	• How are national, state and local policies influencing providers’ organizational behaviors? • What are the effects of these multiple levels of policy/regulation on cities’ health resources? • What tools does local government have to influence health resources? • What is the role of health resources in cities’ urban planning agendas?	• Centers for Medicare and Medicaid Services innovation grants and waivers (federal) • Medicaid design and payment (federal/state) • Licensing, regulation, scope of practice laws (state) • Enforcement of community benefits requirements (state) • “Healthy Cities” commitments (local)
Organizational/health system/provider	• What is the role of private capital on provider organizational behavior? • How do health systems prioritize location and types of services offered? • How do health systems align organizational goals with health equity and access concerns?	• Rent control and rent regulation • Community land trusts • Developer incentives • Landlord tax abatements • Limits on or transparency/disclosure of private equity investment • Local laws directly targeting health system closures and restructuring • Organizational equity and access initiatives
Community/Intrapersonal	• How are public and private health resources utilized within communities, and who utilizes them? • What are the economic effects of health system changes on communities? • What are the employment effects of health systems changes on communities?	• Local hiring and procurement initiatives by health systems and cities • Participatory planning processes for development, zoning, city planning • Tenant and resident organizing movements
Individual	• How do changes in the location and type of care affect residents’ perceptions of their local health resources? • How do changes in location and types of care affect whether residents use or do not use local health resources? What groups are disproportionately affected by these changes?	• Patient education and clinical care initiatives • Patient engagement groups and advisory councils

First, a key aspect of measuring this phenomenon is ensuring that measurements of access to care (which we understand to be the primary outcome of interest) are conceived broadly, considering indicators of equity in access like provider supply, location, types of insurance accepted, waitlists and wait times, and whether providers reflect the racial, cultural and linguistic makeup of the communities they serve. Here we borrow measures of equity in access from those used in research of “universal systems”, conceptualizing equity in access as a supply-side phenomenon that determines whether the services that users need are provided to all who need them [49]. Such measures, when combined with city and neighborhood health data, could help cities identify resource gaps and improve equity in access to care. Importantly, we consider a neighborhood’s health care resources to include a range of private hospitals, physician and specialty practices, urgent care centers, and community and faith-based services, as well as public services like city-operated clinics, and county hospitals. It is the care provided by these institutions together that determine whether residents of a neighborhood have sufficient access to quality health care, but existing research often considers specific types of healthcare services or providers, rather than the full landscape. Definitions of private vs. public services are also not clear-cut. Privately-owned facilities receive public funding in the form of Medicaid or Medicare dollars, federally-funded residency slots, and tax breaks for providing community benefits, while publicly subsidized FQHCs may be operated by private non-profit partners [50]. This makes the impact of public and private investments and policies, key factors in studies of neighborhood gentrification, particularly challenging though important to disentangle. In terms of measuring health care gentrification itself, developing a measure similar to those indexes which have been developed for measuring neighborhood gentrification could help to establish results that are comparative across contexts or places.

We also need to better understand the impact of the multi-level policy and regulatory environments on provider behavior. What are the specific pathways by which public policies and regulations at the national, state and local level drive public and private investments? How are these drivers influencing providers’ organizational behavior, and in turn, the supply of neighborhood health care services? For instance, local governments often lack power and oversight to respond to health care gentrification and its implications despite holding much of the responsibility of ensuring the health of their cities’ residents. While health care delivery systems in the United States straddle the public and private realms, private capital is increasingly prominent. At the state level, health care gentrification may be mediated by policies and programs like Medicaid expansion or waiver programs that make providing care to lower-income individuals a better value proposition. But at a local level, cities have limited tools to influence private health care businesses; they cannot force hospitals to stay open, or private clinics to locate in the areas of highest need. The tools that they do have – control over zoning or tax abatements - do not make up for the low revenue of operating a safety net hospital or clinic, particularly if they continue to lose commercially insured patients. Questions around how state and federal regulations play out at the local level are particularly important when considering the growing role of private equity, whose influence is effectively a black box due to the limited reporting requirements and regulations on many private specialty services. The answers to these questions can help determine key leverage points for action.

Critically, we note that research on health care gentrification must take into account the *lived experience of neighborhood residents*. Currently, many health systems and regions conduct community needs assessments. But these assessments often rely solely on clinical and surveillance data from local health care agencies and providers, without including the perspective of patients and community residents. Resident perspectives, which can be assessed using qualitative methods such as interviews, focus groups, participatory methods such as process mapping, or uniquely designed surveys, not only identify gaps, but help policymakers and providers understand how and why patients are using, or not using, the health system. In our own work, neighborhood residents and health care workers (who are often both users and providers of care in their neighborhoods) are essential key informants.

### Health care gentrification research must also examine solutions

In addition to understanding the multi-level drivers of health care gentrification and its interplay with other urban processes such as neighborhood gentrification, applied research should also focus on finding solutions to reduce and mitigate the impact of health care gentrification, as Thomas et al. describe in their call for a rigorous, action-oriented generation of health equity research [51]. Across the U.S, there are promising avenues and policy levers worthy of further study.

First, federal and state government can use their regulatory power to incentivize providers to increase access for marginalized communities. At the federal level, the Centers for Medicaid and Medicare Services sets Medicare payments and sponsors new payment models, innovation grants and waivers for states such as Medicaid Accountable Health Communities [52]. At the state level, states can use their authority over Medicaid design and payment, licensing, regulation, scope of practice and enforcement of the community benefits non-profit systems are required to provide to drive the allocation of health care resources where they are most needed [50].

While cities may lack the regulatory control of the state and federal government, many are exploring local policies and programs to prevent or control gentrification. Such efforts require collaboration between federal, state and local policymakers, and public and private sectors but can also put more control over services in local hands. For instance, rent control and regulation are city-level measures that have been explored as a way of preventing displacement associated with gentrification. Other options include community land trusts which return control of land and land-use to residents, zoning regulations, developer incentives and tax abatements to incentivize landlords to maintain affordable rents [53]. Similar measures might prevent health care gentrification by supporting community health centers and incentivizing providers to remain in or move to neighborhoods where they are needed. Local policies may also target health care gentrification directly; Philadelphia, for instance, passed a measure to gain more local control over hospital closures by increasing the notice hospitals must give before closing, and requiring them to submit a detailed closure plan addressing continuity of care for patients as well as plans to support laid-off staff, giving the city more leverage to ensure equitable distribution of care [54]. Researchers and policymakers might investigate the short and long-term effects of similar policies through a health care gentrification lens.

Finally, at the organizational level, private health systems also play an important role in meeting the needs of all residents, not just the most profitable, as anchors of neighborhoods and communities. This includes ensuring health care access, working toward addressing social determinants of health, such as housing and quality jobs, and partnering with public providers and state and local governments. For instance, some systems are working on local hiring and procurement initiatives to support residents beyond direct health services (i.e., www.healthcareanchor.network/). These measures could have significant impacts on health and well-being, since health care facilities have long been a local source of stable, middle-class and often unionized jobs.

At a broader scale and highlighting the interdisciplinary nature of health care gentrification, the impacts of planning agendas that employ health promotion rhetoric such as Healthy Cities and Health in All Policies, or promote sustainable and resilient cities using a health motive, should be considered. These trends in policy tend to under-emphasize the role of health care itself arguing that population health is more influenced by other aspects of the environment. But this approach ignores the fact that health care, while not sufficient, is necessary for maintaining population health, particularly considering the needs of chronically ill, elderly, people with disabilities, or pregnant people, among others. Ensuring equitable access to quality health care should thus be purposefully placed within urban planning agendas intended to improve health.

## Conclusions

Ensuring equitable access to health care is a timely concern as the capacity of health care systems has been called into question during the COVID-19 pandemic. Inequitable access to and use of testing, treatment, and vaccination have been evident between and within populations, both in the U.S., the focus of this article, and around the world. The pandemic has also highlighted the role of governance at all levels in protecting population health, and variations in control and politics have resulted in differences in strategies and outcomes. Such observations have been written about broadly. The concept of health care gentrification and research to address inequitable access to quality health care goes beyond the scope of the pandemic, and is relevant to maintaining population health before, during and after such global health emergencies.

## Data Availability

Not applicable.
