# Correction: Advancing urban health equity in the United States in an age of health care gentrification: a framework and research agenda

**DOI:** 10.1186/s12939-022-01686-5

**Published:** 2022-06-16

**Authors:** Helen V. S. Cole, Emily Franzosa

**Affiliations:** 1 Barcelona Lab for Urban Environmental Justice and Sustainability, Institut de Ciencia i Tecnologia Ambientals (ICTA-UAB), Universitat Autonoma de Barcelona, Barcelona, Spain; 2 Healthy Cities research group, Department of Epidemiology and Public Health, Institut Hospital del Mar d’Investigacions Mèdiques (IMIM), Barcelona, Spain; 3 Research Health Science Specialist, Geriatric Research Education and Clinical Center (GRECC), James J. Peters VA Medical Center, Bronx, USA; 4 Brookdale Department of Geriatrics and Palliative Medicine, Icahn School of Medicine at Mount Sinai, New York, USA

**Correction: Int J Equity Health 21, 66 (2022)**

**https://doi.org/10.1186/s12939-022-01669-6**

Following publication of the original article [1], the authors reported a missing statement in the Funding declaration. The following statement has been added to the Funding declaration: ‘This work/research/etc. contributes to the “María de Maeztu” Programme for Units of Excellence of the Spanish Ministry of Science and Innovation (CEX2019–000940-M).’

Furthermore, the article title has been updated to remove “Title:”.

The original article [1] has been updated.
